# The Impact of Patient Deaths on General Surgeons’ Psychosocial Well-Being and Surgical Practices

**DOI:** 10.3389/fsurg.2022.898274

**Published:** 2022-04-28

**Authors:** Cihangir Akyol, Suleyman Utku Celik, Mehmet Ali Koc, Duygu Sezen Bayindir, Mehmet Ali Gocer, Buket Karakurt, Mustafa Kaya, Sena Nur Kekec, Furkan Aydin Simsek

**Affiliations:** ^1^ Department of General Surgery, Ankara University School of Medicine, Ankara, Turkey; ^2^ Department of General Surgery, Gulhane Training and Research Hospital, Ankara, Turkey

**Keywords:** burnout – professional, death & dying, emotional distress, general surgery, well-being, psychosocial support

## Abstract

**Background:**

Patient deaths are an unavoidable occurrence in surgical practice. Although these events have negative effects on patients and their families, they can also have a profound adverse impact on surgeons who are unprepared for these deep emotional experiences. This study aims to investigate the impact of patient deaths on general surgeons’ psychosocial well-being and surgical practices.

**Methods:**

A national cross-sectional survey of a 30-item questionnaire was conducted. The survey evaluated the surgeons’ demographics, professional and practice characteristics, and the impact of patient deaths on their emotional well-being, professional career, and social life.

**Results:**

Four hundred eighty participants completed the survey. One-third of the participants reported that patient deaths affected their emotional well-being, 23.3% reported that patient deaths affected their social life, and 34.2% reported that patient deaths affected their professional career. Surgeons who reported suffering from the emotional impact of death exhibited no differences in terms of place of practice, academic title, surgical experience, work hours, or annual surgical volume. Middle-aged surgeons (*p* = 0.004), females (*p* = 0.041), and surgeons who reported feeling burned out (*p* < 0.001) were more likely to be affected by patient loss. Feelings of sadness, worry, and stress were most reported. A total of 18.1% of the participants indicated that they considered taking a break after patient death, and 11.9% thought they would abandon their surgical career.

**Conclusions:**

The findings of this study suggest that patient death affects surgeons’ psychosocial well-being and surgical practices. Greater awareness and effort are required at the personal, institutional, and organizational level to provide effective support, helping surgeons to cope with the emotional burden of patient deaths.

## Introduction

Adverse events, unexpected complications and patient loss are unavoidable occurrences in surgical practice; therefore, most surgeons will have such experiences at some point in their professional careers (1). Coping with patient death and the families’ associated distress that presents during the course of surgical practice are routinely practiced during a surgeon’s professional career (2). Patient deaths can be emotionally demanding and can negatively affect surgeons’ social and emotional well-being (3–5). During a long career, the overall immediate and long-term effects of these repetitive events, especially if left unaddressed, can be profound, cause posttraumatic stress disorder and burnout, and potentially compromise patient care (6–9).

Few studies have investigated the impact of patient loss on medical students, medical residents, paediatricians, obstetricians, oncologists, or palliative care physicians (10, 11). From a surgical perspective, only limited research has examined the psychological impact of intraoperative adverse events or complications on surgeons (2, 3, 6). Unfortunately, there is no sufficient focus on the impact of perioperative patient death on general surgeons’ behavioural responses, psychosocial well-being, and coping strategies. Today, we know that patient death and the related psychological distress affects professional performance, and mental and physical health (1, 8, 12). In this national cross-sectional survey study, we aim to evaluate surgeons’ emotional and psychosocial reactions to patient loss, and to analyse the relationship between the sociodemographic statuses, professional careers, and surgical practice of the surgeons and their emotional responses.

## Materials and Methods

### Individuals

All members of the Turkish Surgical Society were invited to participate in an online, self-administered survey of the effects deaths on general surgeons’ psychosocial well-being and surgical practices (http://surveymonkey.com) in May 2021. Participation was optional and anonymous. Only general surgeons were included in the study. Residents, nonpractising surgical researchers, and retired surgeons were excluded.

### Questionnaire

A national cross-sectional survey using a 30-item questionnaire was conducted. The survey evaluated the demographics, professional and practice characteristics of the surgeons, and the impact of patient deaths on their emotional well-being, professional career, surgical practice, and social life. For the purpose of this study, patient mortality was defined as any death, regardless of its cause, that occurred during the patient’s treatment course. This definition was shared with all participants at the beginning of the survey.

The questionnaire contained both closed (Likert scale questions) and open-ended questions. For the purpose of analysis, responses of “Never”, “Low”, and “Medium” were considered indicative of disagreeing with the statement, and responses of “High” and “Extremely” were considered as agreeing with the statement. The open-ended last question was about strategies for coping with patient deaths or adverse events. We assessed burnout using a single item that correlates with the emotional exhaustion subscale of the Maslach Burnout Inventory-Human Services Survey for Medical Personnel (13, 14). Participants rated the frequency of burnout on a 7-point Likert scale, with responses ranging from “Never” to “Daily”. Burnout was defined as a high score on the EE and/or DP subscales. The licence for this tool was purchased from Mind Garden (http://www.mindgarden.com).

The survey was pilot tested on five general surgery specialists to ensure the coherence and clarity of its items and for refinement prior to the final survey.

### Data Collection

Responses were collected anonymously through an online survey instrument (SurveyMonkey®) from May 1 to May 31, 2021. Two reminders were sent twice 7 and 14 days after the initial invitation. After 30 days, the survey was closed. An incentive was not offered to surgeons for participating in the study.

### Ethical Approval

This study was approved by the Ankara University School of Medicine Undergraduate Student Research Ethics Committee (approval number: 72189195-050.03.04-E.98889). Completion of the survey was deemed consent to participate.

### Statistical Analysis

Data were extracted from the online database and imported into the Statistical Package for the Social Sciences, version 16.0 (IBM®, Chicago, USA) for analysis. Figures were created using GraphPad Prism, version 8.0 (GraphPad Software Inc, California, USA). Examinations of normal distribution assumptions for continuous variables were visually assessed with quantile-quantile plots and histograms and confirmed with the Shapiro–Wilk test. Normally distributed data were presented as the means ± standard deviations, nonnormally distributed data as medians and ranges, and categorical data as frequencies and percentages. Associations between variables were evaluated using Student’s t test or the Mann–Whitney U test for continuous variables and the Pearson χ^2^ or Fisher exact tests for categorical variables. All tests were two-sided, and a *P* value <0.05 was considered statistically significant.

## Results

A total of 480 general surgeons completed the entire survey, resulting in a response rate of 35.3%. The respondents were predominantly male (*n* = 422, 87.9%), and their mean age was 47.9 ± 10.1 years (range, 28–74 years). The demographic data and occupational characteristics of the respondents are shown in **Table 1**. Nearly half of the surgeons (52.5%) were specialists, and 228 (47.5%) of the surgeons had an academic rank of assistant professors, associate professors, or full professors. A total of 27.3% of the surgeons worked in a training and research hospital, 17.1% worked in a state hospital, 30.0% worked in a university hospital, and 25.6% worked in a private hospital. A total of 354 (73.8%) participants had over 10 years of experience as specialists in general surgery, and their mean experience in surgery was 18.3 ± 11.1 years (range, 1–50 years).

**Table 1:** Sociodemographic and professional characteristics of the general surgeons.

**Characteristics**	** **
Age (years), mean ± SD	47.9 ± 10.1 (range, 28–74)
Sex, *n* (%)	
Female	58 (11.1)
Male	422 (87.9)
Marital status, *n* (%)	
Single	44 (9.2)
Married (or partnered)	436 (90.8)
Academic title, *n* (%)	
Specialist	252 (52.5)
Assistant/Associate professor	134 (27.9)
Professor	94 (19.6)
Workplace, *n* (%)	
Training and research hospital	131 (27.3)
State hospital	82 (17.1)
University hospital	144 (30.0)
Private hospital	123 (25.6)
Experience in surgery (years), mean ± SD	18.3 ± 11.1 (range, 1–50)
Work hours per week, *n* (%)	
<40	76 (15.8)
40–60	364 (75.8)
>60	40 (8.3)
Work hours per week in operating room, *n* (%)	
<10	138 (28.8)
10–20	248 (51.7)
>20	94 (19.6)
Annual surgical volume, *n* (%)	
<50	33 (6.9)
50–150	126 (26.3)
151–250	146 (30.4)
** **>250	175 **(**36.5)

**Figure 1:**
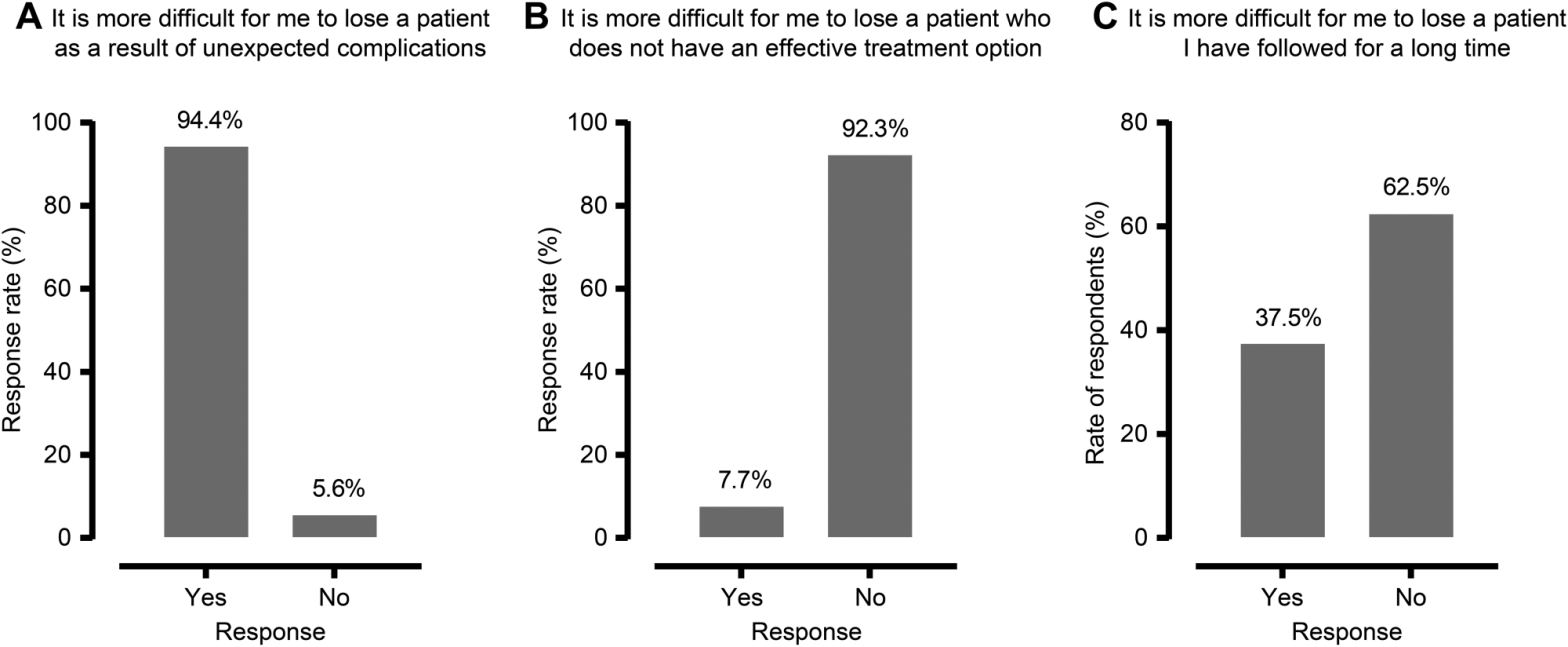
Impact of patient deaths on general surgeons.

One-third of the participants reported that patient deaths affected their emotional well-being, 23.3% reported that their social life was affected, and 34.2% reported that their professional career was affected (**Figure 1**). A total of 18.8% of respondents reported feeling burned out from their work at least once a week. Surgeons who reported suffering from the emotional impact of patient deaths exhibited no differences in terms of age (*p* = 0.767), workplace (*p* = 0.163), academic title (*p* = 0.259), surgical experience (*p* = 0.937), work hours per week (*p* = 0.635), weekly working hours in the operating room (*p* = 0.605), or annual surgical volume (*p* = 0.126). However, female surgeons (*p* = 0.041) and surgeons who reported feeling burned out at least once a week (*p* < 0.001) were found to be more likely to report being negatively affected by a patient loss. Additionally, when age was categorized as <40, 40–59, and ≥60, participants in the middle age group (40–59 years) were found to be significantly more likely to experience emotional exhaustion due to such losses than those in the younger and older groups (*p* = 0.004).

**Figure 2:**
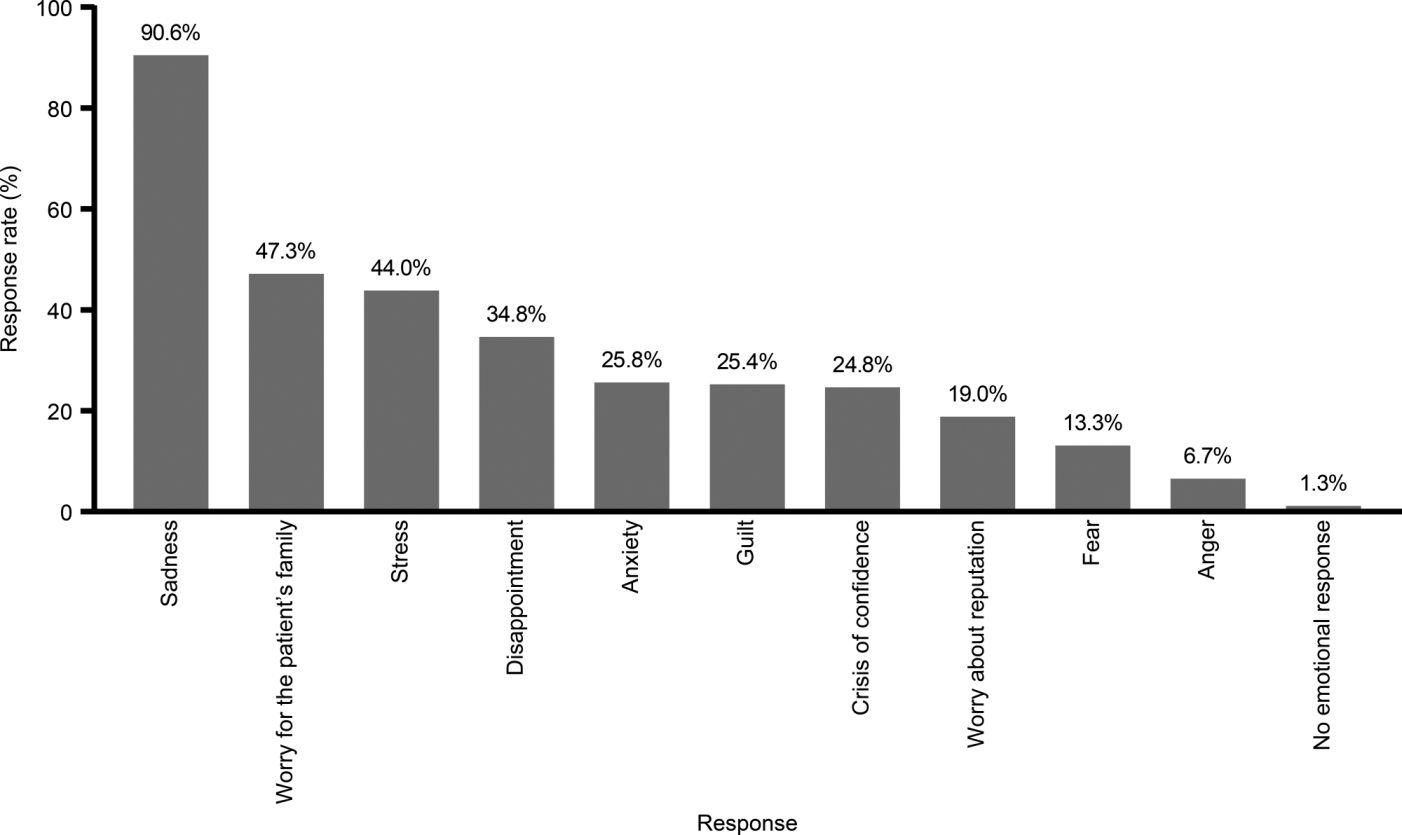
Profiles of responses experienced by general surgeons to patient deaths.

Regarding emotions experienced by surgeons in response to the patient deaths, 90.6% of the participants reported sadness, 47.3% worry for the patients’ family, 44.0% stress, 34.8% disappointment, and 25.8% anxiety about patient loss. Only 1.3% of the participants reported no emotional response to the deaths (**Figure 2**). We also sought to investigate surgeons experiencing repetitive memories after a patient death, having physiological responses to reminders of these events, changing sleeping patterns, and losing interest in enjoyable activities. The most remarkable finding was that the majority of the participants (85.8%) reported experiencing intrusive memories, flashbacks, or dreams that reminded them of the patient’s death, ranging from sometimes to always. The emotional reactions that occurred after patient loss and their frequency are shown in **Table 2**.

**Table 2:** Impact of patient deaths on general surgeons.

	*n* (%)
Experience repetitive memories after a patient death	
Never	68 (14.2)
Sometimes	335 (69.8)
Often	55 (11.5)
Always	22 (4.6)
Physiological responses (palpitations, difficulty in breathing, sweating, etc.) to reminders of events	
Never	280 (58.3)
Sometimes	172 (35.8)
Often	24 (5.0)
Always	4 (0.8)
Losing interest in activities once enjoyed	
Never	229 (47.7)
Sometimes	207 (43.1)
Often	38 (7.9)
Always	6 (1.3)
Change in sleeping pattern (having difficulty in sleeping)	
Never	169 (35.2)
Sometimes	236 (49.2)
Often	58 (12.1)
Always	17 (3.5)

**Figure 3:**
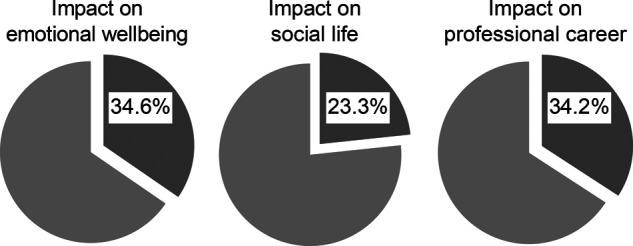
Opinions of the general surgeons about patient losses.

Almost all of the participants (94.4%) stated that it was more difficult to lose a patient because of unexpected complications, 37.5% stated that it was more difficult to lose a patient who was followed for a long time, and 7.7% stated that it was more difficult to lose a patient who had no effective treatment options (**Figure 3**). Additionally, while 31.1% of the participants reported that the patient’s age did not contribute to any negative impact that deaths had on their emotional state, 9.2% reported that the loss of older patients and 59.7% reported that the loss of younger patients was more impressive.

When evaluating the impact of the patient deaths on general surgeons’ professional careers and surgical practices, 18.1% of the participants indicated that they considered taking a break after patient death, 11.9% thought they would abandon their surgical career, and 5.0% thought they would leave their hospital because of the event. A total of 9.8% of the surgeons reported thinking about avoiding surgical procedures for a while and 29.0% reported being less likely to take risks in their surgical practice after experiencing patient death (**Table 3**).

**Table 3:** Impact of patient deaths on general surgeons’ professional careers and surgical practices.

On professional career	
I did not think to give a decision about my career because of the event	333 (69.4)
I thought to take a break for a while	87 (18.1)
I thought to leave my hospital because of the event	24 (5.0)
I thought to give up my surgical career because of the event	57 (11.9)
I thought to give up my professional career because of the event	29 (6.0)
On surgical practice	
My attitude towards my surgical practice has not changed	259 (54.0)
I avoided surgical procedures for a while	47 (9.8)
I avoided taking risks in my surgical practice	139 (29.0)
I tried to improve my surgical skills	158 (32.9)

Regarding social support or negative social exchange, 41.7% of the participants reported receiving support from their families, 37.9% from their peers, 35.4% from their senior colleagues, and 3.1% from their institutions; 1.9% stated that they received psychological counselling. After patient deaths, 48.1% of the surgeons reported receiving negative reactions from patients’ relatives, 6.3% from other surgeons, and 2.3% from their institutions.

Participants’ suggestions for supporting surgeons in such cases are as follows: education on coping with patient death (51.5%), formal mentoring system (31.3%), psychological therapy counselling (27.7%), peer colleague support groups (27.3%), a time break after a patient death (13.5%), and morbidity/mortality meetings (11.5%) (**Table 4**).

**Table 4:** Suggestions of the general surgeons for coping with patient losses or adverse events.

Suggestions	*n* (%)
Education on coping with patient deaths	247 (51.5)
Formal mentoring system	150 (31.3)
Psychological therapy counselling	133 (27.7)
Peer colleague support groups	131 (27.3)
A time break after a patient loss	65 (13.5)
Morbidity and mortality meetings	55 (11.5)
Legal support	30 (6.3)

## Discussion

This national survey suggests that patient deaths have significant effects on general surgeons. Although patients and their families were mostly affected by errors, adverse events, postoperative complications, or deaths, surgeons may be the “second victims” in such circumstances (4, 5, 15, 16). In this study, up to one-third of the surgeons reported that patient deaths affected their emotional well-being, social life, or professional career. Nearly one-fifth considered taking a break from work for a while and nearly one in 10 surgeons considered giving up their surgical career because of psychological distress of perioperative patient loss. These findings show that surgeons experience strong emotional and social reactions to these undesirable events. Commonly reported emotional difficulties were sadness, worry, stress, disappointment, anxiety, and guilt after patient loss.

The complex, invasive, and potentially risky nature of surgical treatment substantially shapes the relationship between a patient and his or her surgeon and requires an increased level of confidence and trust from the patient (17). Additionally, this unspoken “contract” may make adverse events, errors, and patient death even more emotionally challenging for surgeons than in other clinical settings and, therefore, creates a major psychological burden (5, 16, 18). To date, limited studies investigated the impact of patient death on surgeons and their reactions to such events. Patient loss showed significant negative psychosocial consequences (1, 3, 4, 6, 19). These include feelings of guilt and failure, decreased effectiveness, poor decision-making, depression, burnout, and posttraumatic stress disorder (1, 18–20). This study showed that female surgeons and those who reported feeling burned out were more likely to report being emotionally negatively affected by patient deaths. Additionally, middle-aged (40–59 years) surgeons were significantly more likely to experience strong emotional responses due to such losses than those in the younger and older groups, which contradicts the findings of previous studies that found a positive correlation between years of professional experience and reduced emotional engagements with dying patients and bereaved families (21). This may be associated with increased years of clinical practice that allow surgeons to identify with patients more easily and to become more emotionally involved. Multiple factors may contribute to the psychological impact of a patient death on a surgeon. In a systematic review by Joliat et al. (1), unexpected deaths were found to adversely affect surgeons’ minds more than patients with life-limiting illnesses, as demonstrated in this study. A qualitative study by Zambrano et al. (18), which interviewed nine Australian surgeons, suggested that surgical oncologists were less emotionally distant from patients. This means that these surgeons had more positive emotions regarding patient loss than other physicians who established clear limits and who were more likely to consider death as defeat instead of a natural progression. In this study, variables such as academic title, workplace, surgical experience, work hours, and surgical volume did not show a significant impact on emotional responses after patient death. Such stressful events affect the social and emotional well-being of surgeons and negatively affect their surgical practice and professional career. In a study, Goldstone et al. (22) investigated whether an intraoperative death had an adverse effect on operation outcomes within 48 h after the event. The study revealed that patients undergoing surgery within 48 h after the surgeon experienced an intraoperative death of a patient had longer intensive care unit and hospital lengths of stay. Moreover, concerns related to possible medicolegal issues about patient deaths may lead surgeons to practice defensive medicine. It exposes patients to the risk of unnecessary diagnostic testing and imaging, consultations, or exclusion from treatments and increases in health care expenditures (5, 23, 24). This finding indicates that the professional performance of a surgeon, patient outcomes and health care costs may, indeed, be negatively affected by patient deaths.

Adequate training, debriefings, and formal instructions are important to cope with guilt and not to negatively affect the psychosocial well-being of physicians after patient deaths (3, 25). In a study, Gold et al. (26) showed that physicians who were adequately trained to cope with a patient death were reported to be less emotionally affected and to feel less guilty for a death. Despite the expectation that physicians will learn to cope with the emotional burden of dealing with death, they have continuously faced challenges to well-being during the course of their careers (19). The findings of this study showed that surgeons highly regarded support and advice from their peers. In addition, they reported the need to make more organizational changes and improvements aimed at supporting and training them to deal with the stress of patient deaths. Common suggestions included education on coping with these events, effective formal mentorship for young surgeons, peer colleague support groups for senior surgeons, morbidity and mortality meetings for the discussion of complications, and legal support. Additionally, a time break from the operation after patient loss was also suggested by some surgeons. Only 3.1% received support from their institutions, and 1.9% of surgeons received professional psychological counselling. Up to one-third of the surgeons preferred informal sources of support, such as family, peers, and senior colleagues; however, these may not be sufficient for adequately managing the emotional burden of a patient loss. This result reveals the necessity of organizational, institutional, and national efforts to promote the well-being of surgeons and the quality of care that patients receive. In some studies of “support programs”, based on concepts related to emotional support and to foster grief processing, researchers established a formal peer support and mentoring program in an effort to aid surgeons in coping with adverse events, errors, and patient death (15, 27). Such surgery-specific support programs and strategies have the potential not only to support surgeon well-being but also to promote patient safety and to reduce burnout and posttraumatic distress. This could be because colleagues were likely to have been in similar circumstances and to have experienced similar emotions in the past. Therefore, these programs should also be incorporated into medical and postgraduate surgical education. However, the development and dissemination of these efforts are needed.

This study has the limitations inherent in survey research. These data are cross-sectional and subject to sampling error, bias due to the self-reported nature of the data, and unmeasured confounding. There is also the possibility of selection bias. Those who responded to the survey may be more interested in, or affected by, issues such as emotional distress and patient death compared to their counterparts who chose not to respond. However, it is the largest nationwide study to date reporting the burden of dealing with patient death specifically among general surgeons ever conducted.

## Conclusion

Complications and patient deaths are unavoidable parts of surgical practice. Repeated exposure to such events in an unsupported clinical environment may lead to negative emotional consequences. The findings of this study indicate that patient loss negatively impacts surgeons’ psychosocial well-being and professional career. Therefore, efforts to identify at-risk populations, develop effective strategies to cope with the stress of patient deaths and improve access to support systems are needed. We would like to conclude this article with a quotation from a vascular surgeon René Leriche’s *La Philosophie de la chirurgie* (1958): “Every surgeon carries within himself a small cemetery, where from time to time he goes to pray—a place of bitterness and regret, where he must look for an explanation for his failures.”

## Data Availability

The raw data supporting the conclusions of this article will be made available by the authors, without undue reservation.

## References

[1] Joliat GR Demartines N Uldry E. Systematic review of the impact of patient death on surgeons. Br J Surg. (2019) 106:1429–32. 10.1002/bjs.11264 31373690

[2] Turner K Johnson C Thomas K Bolderston H McDougall S. The impact of complications and errors on surgeons. Bull R Coll Surg Engl. (2016) 98:404–7. 10.1308/rcsbull.2016.404

[3] Pinto A Faiz O Bicknell C Vincent C. Surgical complications and their implications for surgeons’ well-being. Br J Surg. (2013) 100:1748–55. 10.1002/bjs.9308 24227360

[4] Bohnen JD Lillemoe KD Mort EA Kaafarani HMA. When things go wrong: the surgeon as second victim. Ann Surg. (2019) 269:808–9. 10.1097/sla.0000000000003138 30480564

[5] Siddaiah-Subramanya M To H Haigh C. The psychosocial impact of surgical complications on the operating surgeon: a scoping review. Ann Med Surg (Lond). (2021) 67:102530. 10.1016/j.amsu.2021.102530 34276982 PMC8267492

[6] Srinivasa S Gurney J Koea J. Potential consequences of patient complications for surgeon well-being: a systematic review. JAMA Surg. (2019) 154:451–7. 10.1001/jamasurg.2018.5640 30916741

[7] Celik SU Aslan A Coskun E Coban BN Haner Z Kart S Prevalence and associated factors for burnout among attending general surgeons: a national cross-sectional survey. BMC Health Serv Res. (2021) 21:39. 10.1186/s12913-020-06024-5 33413318 PMC7792210

[8] Thompson CV Naumann DN Fellows JL Bowley DM Suggett N. Post-traumatic stress disorder amongst surgical trainees: an unrecognised risk? Surgeon. (2017) 15:123–30. 10.1016/j.surge.2015.09.002 26482084

[9] Lillemoe HA Geevarghese SK. Stopping the progression of moral injury: a priority during surgical training. Ann Surg. (2021) 274:e643–5. 10.1097/sla.0000000000005153 34387198

[10] Kelly E Nisker J. Medical students’ first clinical experiences of death. Med Educ. (2010) 44:421–8. 10.1111/j.1365-2923.2009.03603.x 20236239

[11] Granek L Ben-David M Nakash O Cohen M Barbera L Ariad S Oncologists’ negative attitudes towards expressing emotion over patient death and burnout. Support Care Cancer. (2017) 25:1607–14. 10.1007/s00520-016-3562-y 28084531

[12] Duncan SE Arnon R DiPietrantonio C Ehrlich K Knight CS Chu J Pediatric liver transplant teams coping with patient death. J Pediatr Gastroenterol Nutr. (2018) 67:169–72. 10.1097/mpg.0000000000001991 29620594

[13] West CP Dyrbye LN Sloan JA Shanafelt TD. Single item measures of emotional exhaustion and depersonalization are useful for assessing burnout in medical professionals. J Gen Intern Med. (2009) 24:1318–21. 10.1007/s11606-009-1129-z 19802645 PMC2787943

[14] Maslach C Jackson SE Leiter MP. Maslach burnout inventory: Manual. Menlo Park, CA: Mind Garden, Inc (2018).

[15] El Hechi MW Bohnen JD Westfal M Han K Cauley C Wright C Design and impact of a novel surgery-specific second victim peer support program. J Am Coll Surg. (2020) 230:926–33. 10.1016/j.jamcollsurg.2019.10.015 31857209

[16] Han K Bohnen JD Peponis T Martinez M Nandan A Yeh DD The surgeon as the second victim? Results of the Boston intraoperative adverse events surgeons’ attitude (BISA) study. J Am Coll Surg. (2017) 224:1048–56. 10.1016/j.jamcollsurg.2016.12.039 28093300

[17] Axelrod DA Goold SD. Maintaining trust in the surgeon-patient relationship: challenges for the new millennium. Arch Surg. (2000) 135:55–61. 10.1001/archsurg.135.1.55 10636348

[18] Zambrano SC Chur-Hansen A Crawford GB. How do surgeons experience and cope with the death and dying of their patients? A qualitative study in the context of life-limiting illnesses. World J Surg. (2013) 37:935–44. 10.1007/s00268-013-1948-2 23417623

[19] Zambrano SC Chur-Hansen A Crawford GB. On the emotional connection of medical specialists dealing with death and dying: a qualitative study of oncologists, surgeons, intensive care specialists and palliative medicine specialists. BMJ Support Palliat Care. (2012) 2:270–5. 10.1136/bmjspcare-2012-000208 24654200

[20] Oskrochi Y Maruthappu M Henriksson M Davies AH Shalhoub J. Beyond the body: a systematic review of the nonphysical effects of a surgical career. Surgery. (2016) 159:650–64. 10.1016/j.surg.2015.08.017 26431813

[21] Peisah C Latif E Wilhelm K Williams B. Secrets to psychological success: why older doctors might have lower psychological distress and burnout than younger doctors. Aging Ment Health. (2009) 13:300–7. 10.1080/13607860802459831 19347697

[22] Goldstone AR Callaghan CJ Mackay J Charman S Nashef SA. Should surgeons take a break after an intraoperative death? Attitude survey and outcome evaluation. BMJ. (2004) 328:379. 10.1136/bmj.37985.371343.EE 14734519 PMC341385

[23] Pellino IM Pellino G. Consequences of defensive medicine, second victims, and clinical-judicial syndrome on surgeons’ medical practice and on health service. Updates Surg. (2015) 67:331–7. 10.1007/s13304-015-0338-8 26650202

[24] Bourne T Shah H Falconieri N Timmerman D Lees C Wright A Burnout, well-being and defensive medical practice among obstetricians and gynaecologists in the UK: cross-sectional survey study. BMJ Open. (2019) 9:e030968. 10.1136/bmjopen-2019-030968 31767585 PMC6887071

[25] Serou N Sahota L Husband AK Forrest SP Moorthy K Vincent C Systematic review of psychological, emotional and behavioural impacts of surgical incidents on operating theatre staff. BJS Open. (2017) 1:106–13. 10.1002/bjs5.21 29951612 PMC5989958

[26] Gold KJ Kuznia AL Hayward RA. How physicians cope with stillbirth or neonatal death: a national survey of obstetricians. Obstet Gynecol. (2008) 112:29–34. 10.1097/AOG.0b013e31817d0582 18591304

[27] Busch IM Moretti F Campagna I Benoni R Tardivo S Wu AW Promoting the psychological well-being of healthcare providers facing the burden of adverse events: a systematic review of second victim support resources. Int J Environ Res Public Health. (2021) 18:5080. 10.3390/ijerph18105080 34064913 PMC8151650

